# The Ratio of Low-Frequency to High-Frequency in Ambulatory Electrocardiographic Monitoring Immediately Before Coronary Angiography as a Predictor of the Presence of Coronary Artery Disease

**DOI:** 10.4021/jocmr1661w

**Published:** 2013-12-13

**Authors:** Yuiko Miyase, Shin-ichiro Miura, Yuhei Shiga, Ayumi Nakamura, Kenji Norimatsu, Hiroaki Nishikawa, Keijiro Saku

**Affiliations:** a Department of Cardiology, Fukuoka University School of Medicine, Fukuoka 814-0180, Japan; b Department of Molecular Cardiovascular Therapeutics, Fukuoka University School of Medicine, Fukuoka 814-0180, Japan

**Keywords:** Coronary artery disease, Autonomic nervous system, Heart rate variability, Neuropeptides

## Abstract

**Background:**

There is considerable evidence that impaired autonomic control may be associated with the etiology of coronary artery disease (CAD). We hypothesized that the autonomic imbalance as assessed by measuring heart rate variability (HRV) and biological parameters before and after coronary angiography (CAG) may predict the presence of CAD.

**Methods:**

Ambulatory electrocardiographic (ECG) examination using eHEART^®^ (Parama-Tec) is a novel, rapid, and simple method with which we can measure HRV within 5 min. We selected patients (n = 78, 68 ± 10 y) who underwent CAG and analyzed their ambulatory ECGs and blood levels of neuropeptides at both 1 day and immediately before and after CAG. The patients were divided into the presence (n = 64, CAD group) and absence of CAD (n = 14, non-CAD group).

**Results:**

Although the CAD group showed an increase in blood pressure immediately before CAG, the ratio of low-frequency to high-frequency (LF/HF) was significantly decreased in the CAD group, but not in the non-CAD group. On the other hand, there was no difference in a coefficient of variation of the R-R interval or pulse rate between the two groups. CAD was independently associated with hypertension (P = 0.011), dyslipidemia (P = 0.009), and LF/HF immediately before CAG (P = 0.046) by a logistic regression analysis.

**Conclusions:**

These findings suggest that LF/HF immediately before CAG in addition to hypertension and dyslipidemia might predict the presence of CAD.

## Introduction

There is considerable evidence that impaired cardiac autonomic control may be associated with the etiology of coronary artery disease (CAD) [1, 2]. Impaired heart rate variability (HRV) reflects cardiac sympathetic and parasympathetic modulation [3], and can provide information on the progression of focal coronary atherosclerosis beyond that obtained with traditional risk markers of atherosclerosis [4]. A population-based, prospective study suggested that altered cardiac autonomic activity, especially lower parasympathetic activity, is associated with the risk of developing CAD [5]. In addition, impaired HRV has also been associated with hypertension (HT), diabetes mellitus (DM), mortality after myocardial infarction, and sudden cardiac death [6-8].

Ambulatory electrocardiographic (ECG) monitoring for 24 h is generally used to assess HRV. Recently, however, an ambulatory ECG examination using eHEART^®^ (Parama-Tec, Fukuoka, Japan) has become available for clinical use. This equipment offers a novel, rapid, and simple method with which we can measure HRV, such as the coefficient of variation of the R-R interval (CVRR), high-frequency (HF), low-frequency (LF), and the ratio of LF to HF (LF/HF), all within 5 min.

Autonomic control can also be evaluated by measuring blood levels of catecholamines (adrenaline (Ad), noradrenaline (NAd), and dopamine (DOA)) and neuropeptides, such as neuropeptide Y (NPY), pancreatic polypeptide (PP), and urinary L-type fatty acid binding protein (U-L-FABP). Catecholamines are highly sensitive to sympathetic activity [9], and PP reflects parasympathetic activity of the vagus nerve [10]. Patients with acute coronary syndrome (ACS) have high U-L-FABP levels, and U-L-FABP measurements may be useful for identifying patients who are at high risk for future cardiovascular events after ACS [11].

Patients with suspected CAD need to receive coronary angiography (CAG) for the diagnosis of CAD, and CAG may affect the autonomic imbalance. We hypothesized that the autonomic response before and after CAG may predict the presence of CAD. Therefore, we enrolled patients who underwent CAG and analyzed their ambulatory ECG and blood levels of neuropeptides and U-L-FABP at both 1 day and immediately before and after CAG.

## Methods

### Study population

From April 2012 to August 2012, we enrolled 115 consecutive patients who underwent CAG because of suspected CAD at Fukuoka University Hospital. After ECG examination, we checked the 5 min ECG recording in all patients. When the patients showed a poor ECG recording and had arrhythmias in more than 10% of their recordings, we excluded the patients from the study. The eHEART automatically calculated HRV after we manually deleted the ECG recording with arrhythmias. Thus, we excluded 37 patients including the patients who failed to provide blood and urine samples. Finally, we analyzed 78 patients and divided the patients into CAD (n = 64) and non-CAD (n = 14) groups. The presence of CAD was defined according to significant coronary stenosis (> 50%, at least one coronary vessel) by CAG. The protocol in this study was approved by the ethics committee of Fukuoka University Hospital, and all subjects gave their written informed consent to participate. We collected and analyzed all data using the medical database of Fukuoka University Hospital.

### Evaluation of coronary risk factors

We compared the CAD and non-CAD groups with regard to coronary risk factors based on fasting serum samples. Coronary risk factors included age, gender, obesity (body mass index (BMI)), HT, dyslipidemia (DL), DM, hyperuricemia (HU), smoking, and chronic kidney disease (CKD). Patients who had a current systolic blood pressure (SBP)/diastolic BP (DBP) ≥ 140/90 mmHg or who were receiving antihypertensive therapy were considered to have HT. DM was defined using the Japan Diabetes Society Criteria or if the patient was being treated with an oral hypoglycemic agent or insulin. Patients with LDL-C ≥ 140 mg/dL, TG ≥ 150 mg/dL, and/or HDL-C < 40 mg/dL, or who were receiving lipid-lowering therapy, were considered to have DL. Obesity was defined as a BMI > 25 kg/m^2^. Medications included β-blocker, α-blocker, αβ-blocker, calcium channel blocker (CCB), angiotensin II receptor blocker (ARB), angiotensin converting enzyme inhibitor (ACE-I), and diuretic.

### Measurement of BP, HR, and HRV

Beat-to-beat HR data in the supine positions were continuously recorded for 5 min using eHEART^®^ at 4 time-points (at both 1 day and immediately before and after CAG). We defined the time-point at 1 day before CAG as the baseline. We evaluated HRV, such as CVRR, HF, LF, and LF/HF. The fluctuations of R-R intervals were integrated on the HF band (0.15 - 0.40 Hz) and the LF band (0.05 - 0.15 Hz). HRV was expressed as the power of the LF and HF components and the LF/HF power ratio. Supine BP and HR were also measured at these 4 points.

### Measurement of blood and urinary biomarkers

Blood levels of catecholamines (Ad, NAd, and DOA), NPY, PP, N terminal-pro brain natriuretic peptide (NT-proBNP), and U-L-FABP were measured at 2 points (at 1 day before and after CAG). All blood and urinary samples were drawn after the patients had fasted overnight. The concentrations of NPY and PP in plasma and L-FABP in urine were determined in duplicate by specific enzyme immunoassays (R&D Systems, Minneapolis, MN, USA) according to the manufacturer’s instructions. At our laboratory, the intra- and inter-assay coefficients of variation were each < 5%.

### Statistical analysis

Statistical analysis was performed using the Stat View statistical software package (Stat View 5; SAS Institute Inc., Cary, NC, USA). Data are expressed as the mean ± standard deviation (SD). The significance of differences was evaluated using the unpaired and paired t-test for continuous variables and the χ^2^ test for non-continuous variables. Multivariate analysis was performed using a logistic regression analysis for independent variables that were related to the presence or absence of CAD. A value of P < 0.05 was considered significant.

## Results

### Patient characteristics

Table 1 shows the baseline clinical characteristics in the CAD and non-CAD groups. In the CAD group, the percentages (%) of HT, DL, and DM were 97%, 88%, and 45%, respectively, and % HT and % DL were significantly higher than those in the non-CAD group. In addition, the incidence of ARB treatment in the CAD group was significantly higher than that in the non-CAD group, whereas there were no differences in BP, HR, left ventricular ejection fraction (LVEF), eGFR or blood levels of NT-proBNP (Table 2).

**Table 1:** Baseline Clinical Characteristics in the CAD and Non-CAD Groups

	All	CAD (n = 64)	Non-CAD (n = 14)
Age (years)	68 ± 10	68 ± 10	65 ± 13
Male, n (%)	53 (68)	45 (70)	8 (57)
BMI (kg/m^2^)	24.0 ± 3.8	24.0 ± 3.6	24.1 ± 4.8
HT, n (%)	71 (91)	62 (97)*	9 (64)
DL, n (%)	63 (81)	56 (88)*	7 (50)
DM, n (%)	33 (42)	29 (45)	4 (29)
HU, n (%)	10 (13)	9 (14)	1 (7)
CKD, n (%)	34 (44)	30 (47)	4 (29)
Smoking, n (%)	45 (58)	38 (59)	7 (50)
Sedation, n (%)	18 (23)	14 (22)	4 (29)
Medication			
β-blocker, n (%)	7 (9)	6 (9)	1 (7)
α-blocker, n (%)	2 (3)	2 (3)	0 (0)
αβ-blocker, n (%)	13 (17)	12 (19)	1 (7)
CCB, n (%)	54 (69)	45 (70)	9 (64)
ARB, n (%)	45 (71)	41 (59)*	4 (29)
ACE-I, n (%)	9 (12)	7 (11)	2 (14)
Diuretic, n (%)	17(22)	4(22)	3(21)

BMI, body mass index; HT, hypertension; DL, dyslipidemia; DM, diabetes mellitus; HU, hyperuricemia; CKD, chronic kidney disease; CCB, calcium channel blocker; ARB, angiotensin II receptor blocker; ACE-I, angiotensin converting enzyme inhibitor. *P < 0.05 vs. non-CAD.

**Table 2:** Various Parameters in the CAD and Non-CAD Groups

	All	CAD	Non-CAD
SBP (mmHg)	128 ± 15	129 ± 16	123 ± 10
DBP (mmHg)	71 ± 10	71 ± 10	73 ± 11
HR (/min)	73 ± 12	72 ± 11	75 ± 16
EF (%)	62 ± 8	62 ± 8	63 ± 5
eGFR (mL/min)	63 ± 17	62 ± 17	67 ± 17
NT-proBNP (pg/mL)	182 ± 257	205 ± 278	80 ± 64

SBP, systolic blood presure; DBP, diastolic blood presure; HR, heart rate; EF, ejection fraction; eGFR, estimated glomerular filtration rate; NT-proBNP, N terminal B-type natriuretic peptide. There were no significant differences in these parameters between CAD and non-CAD.

### Time-course of BP and HR

**Figure 1:**
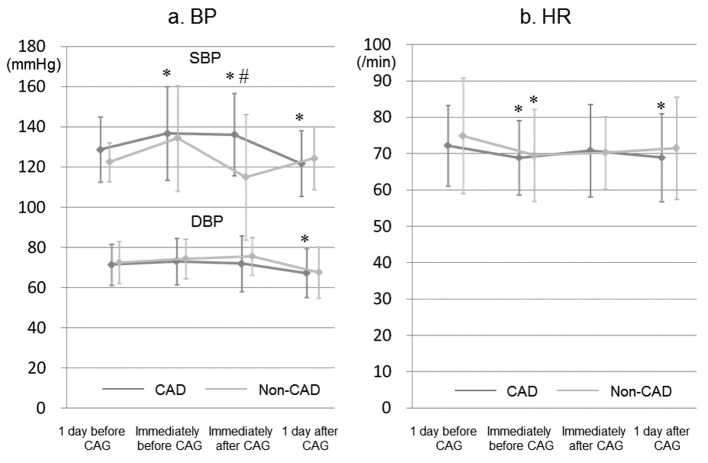
Time-course of BP and HR at both 1 day and immediately before and after CAG in the CAD and non-CAD groups. SBP, systolic blood pressure; DBP, diastolic blood pressure; HR, heart rate. *P < 0.05 vs. at 1 day before CAG (baseline). #P < 0.05 vs. non-CAD group.

As shown in Figure 1, in the CAD group, SBPs immediately before and after CAG were significantly higher than that at baseline. SBP at 1 day after CAG was significantly lower in the CAD group, whereas there were no significant changes in BP in the non-CAD group. In addition, HR immediately before CAG in both groups was significantly reduced compared to that at baseline, as was HR at 1 day after CAG in the CAD group.

### Time-course of LF, HF, LF/HF, and CVRR

**Figure 2:**
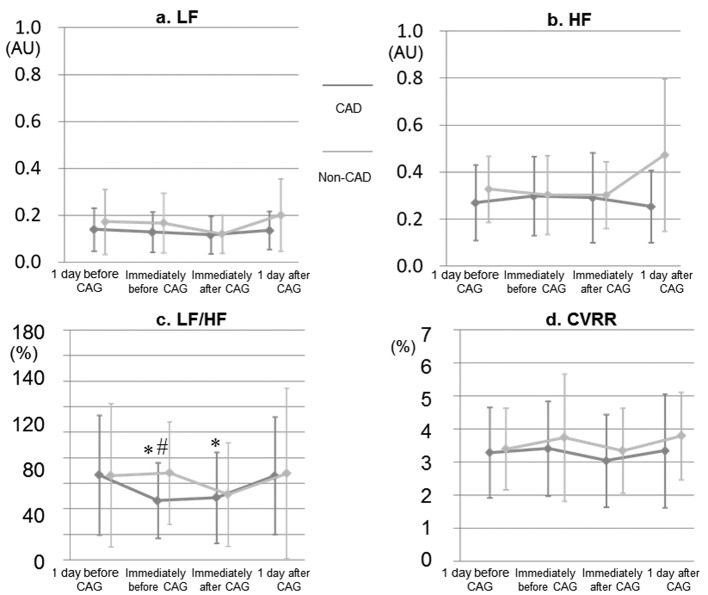
Time-course of LF, HF, LF/HF, and CVRR at both 1 day and immediately before and after CAG in the CAD and non-CAD groups. LF, low-frequency; HF, high-frequency; LF/HF, ratio of LF to HF; CVRR, coefficient of variation of the R-R interval; AU, arbitrary unit. *P < 0.05 vs. at 1 day before CAG (baseline). #P < 0.05 vs. non-CAD group.

Figure 2 shows each parameter of HRV. There were no significant changes in LF, HF or CVRR in the CAD and non-CAD groups, except for significant increases in LF and HF at 1 day after CAG in the CAD group. Interestingly, LF/HF immediately before and after CAG was significantly lower than that at baseline in the CAD group. In addition, LF/HF immediately before CAG in the CAD group was significantly lower than that in the non-CAD group.

### Changes in blood and urinary biomarkers

**Figure 3:**
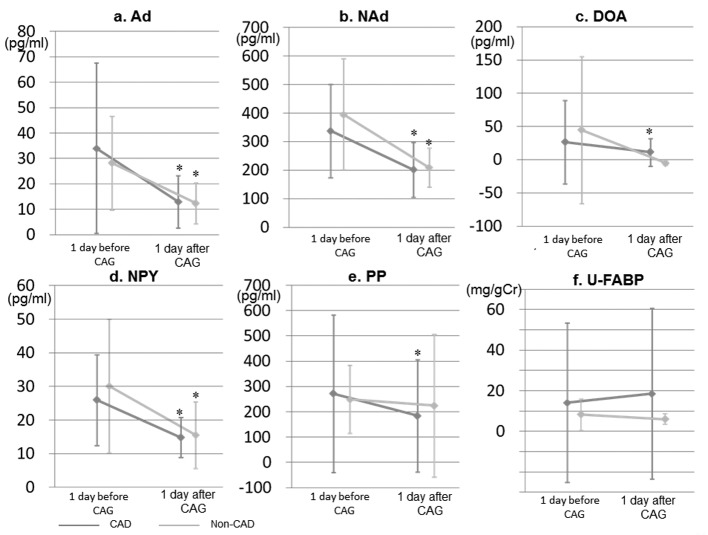
Changes in blood and urinary biomarkers at 1 day before and after CAG in the CAD and non-CAD groups. Ad, adrenaline; NAd, noradrenaline; DOA, dopamine; NPY, neuropeptide Y; PP, pancreatic polypeptide; U-L-FABP, urinary L-type fatty acid binding protein. *P < 0.05 vs. at 1 day before CAG (baseline).

Blood levels of Ad, NAd, and NPY were significantly reduced at 1 day after CAG in the CAD and non-CAD groups (Fig. 3). Although blood levels of DOA and PP were significantly decreased at 1 day after CAG in the CAD group, there were no differences in the changes in DOA and PP between the groups. In addition, there were no changes in U-L-FABP in either group.

### Predictor of the presence of CAD

Next, we analyzed predictors of the presence of CAD using independent variables by a logistic regression analysis (Table 3). We selected HT, DL, and LF/HF immediately before and after CAG in addition to age, gender, and BMI as independent variables, since % HT and % DL in the CAD group were significantly higher than those in the non-CAD group, and since the LF/HF values immediately before and after CAG were significantly lower than that at baseline in the CAD group. CAD was independently associated with HT (P = 0.011), DL (P = 0.009), and LF/HF immediately before CAG (P = 0.046).

**Table 3:** Predictors of the Presence of CAD

Factors	OR (95% CI)	P value
Age	1.06 (0.98-1.15)	0.172
Male	0.46 (0.08-2.65)	0.387
BMI	0.91 (0.73-1.13)	0.388
HT	22.7 (2.03-254)	0.011
DL	12.3 (1.89-80.7)	0.009
LF/HF (immediately before CAG)	0.98 (0.95-1.00)	0.046
LF/HF (immediately after CAG)	1.01 (0.99-1.03)	0.495

BMI, body mass index; HT, hypertension; DL, dyslipidemia; LF/HF, the ratio of low-frequency to high-frequency; CAG, coronary angiography.

## Discussion

First, we found that LF/HF immediately before CAG was significantly decreased in the CAD group, but not in the non-CAD group, although the CAD group showed an increase in BP. Second, CAD was independently associated with HT, DL, and LF/HF immediately before CAG.

The presence of CAD was independently associated with LF/HF immediately before CAG, in addition to HT and DL. It is reasonable that CAD was independently associated with HT and DL in this study because it is well known that HT and DL are critical risk factors for the onset and progression of CAD [12]. Surprisingly, lower LF/HF immediately before CAG was also shown to be a predictor. In this study, there was no difference in % patients with sedation during CAG between the CAD and non-CAD groups, as shown in Table 1. Most previous reports have indicated that HF, LF, and LF/HF were not associated with the onset and progression of CAD [5, 13]. On the other hand, the progression of coronary atherosclerosis was independently predicted by the standard deviation of all normal-to-normal R-R intervals (SDNN) [4]. CCV_RSA_ (CCV, coefficient of component variance; RSA, respiratory sinus arrhythmia) has been shown to be significantly decreased with an increase in coronary angiographic severity [2]. Thus, SDNN and CCV_RSA_, but not LF/HF, were associated with CAD according to previous reports. Moreover, since lower LF/HF was seen immediately before CAG in the CAD group, we considered the autonomic control immediately before and after CAG in both the CAD and non-CAD patients. The CAD group showed CAG-induced elevation of BP immediately before and after CAG, whereas there were no changes in BP in the non-CAD group. The percent of HT in the CAD group was significantly higher than that in the non-CAD group and the sympathetic nervous system both promotes and amplifies the hypertensive state [14]. Since the CAD group showed a reduction in LF/HF (which may be a measure of sympathetic activity) immediately before and after CAG, this reduction may have reflected an attempt to lower BP.

In this study, beat-to-beat HR data were continuously recorded for 5 min using eHEART^®^. This equipment is convenient and both rapid and simple to use, and 5 min of recording was sufficient for an analysis. A previous report indicated that 5 min was sufficient for short-term HRV analysis [15]. In addition, Bigger et al recommended that HF power should be based on at least 1 min of recording and LF power required at least 2.5 min [16]. In fact, Liao et al reported that lower HRV was associated with the development of CAD in patients with DM using 2 min of beat-to beat RR interval data [5].

Although blood levels of catecholamine and neuropeptide were significantly decreased at 1 day after CAG, the levels in the CAD group did not differ from those in the non-CAD group. However, the differences between the groups were too small to cause a corresponding difference in sympathetic and/or parasympathetic activities. The blood levels significantly decreased after CAG, whereas LF, HF, and LF/HF at baseline were similar to those at 1 day after CAG. Changes in catecholamine and neuropeptide may not directly influence LF, HF, or LF/HF. In fact, catecholamine increased and LF/HF decreased at day 1 after the operation in a study on the role of the autonomic nervous system in the development of atrial fibrillation after coronary artery bypass surgery [17]. In addition, since we did not analyze the blood levels immediately before and after CAG, we might not have observed differences in changes in the levels between the CAD and non-CAD groups.

This study has several limitations. First, the sample size was relatively small and this study was cross-sectional, so causal conclusions can be drawn. Second, the HRV measurements were performed under various medications. Although many of the patients were taking medications that may have influenced the measurements, we analyzed time-course of HRV in each patient and analyzed changes of HRV before and after CAG. In addition, there were no differences in % α-blocker, % β-blocker and % αβ-blocker between non-CAD and CAD groups, although sympathetic blocking agents influence HRV in particular. Third, respiratory rate (RR) may also influence the measurement, although we did not count RR. Fourth, the non-CAD group described in this study was a special group and was not considered to be a usual control group, since the patients underwent CAG because of suspected CAD. Prospective studies are needed to clarify the associations we identified.

### Conclusions

LF/HF immediately before CAG in addition to HT and DL might predict the presence of CAD, although the mechanism of the association between the increase in BP and the decrease in LF/HF is not yet clear.
